# Lack of functional KL-VS polymorphism of the *KLOTHO* gene in the Korean population

**DOI:** 10.1590/1678-4685-GMB-2015-0160

**Published:** 2016-06-16

**Authors:** Hee-Kwon Kim, Byung-Hoon Jeong

**Affiliations:** 1 Korea Zoonosis Research Institute, Chonbuk National University, Iksan, Jeonbuk, Republic of Korea; 2 Department of Nuclear Medicine, Molecular Imaging & Therapeutic Medicine Research Center, Biomedical Research Institute, Chonbuk National University Medical School and Hospital, Jeonju, Republic of Korea; 3 Department of Bioactive Material Sciences, Chonbuk National University, Jeonju, Jeonbuk Republic of Korea

**Keywords:** Aging, *KLOTHO* gene, KL-VS polymorphism, population genetics

## Abstract

The functional variant of the Klotho "KL-VS" stretch, which includes six polymorphisms in linkage disequilibrium, is reportedly associated with healthy aging and longevity in European and American populations. Among Asian populations, this variant has been observed in the Indian population but not in the Iranian population. An association between KL-VS polymorphism and aging has not been reported in Koreans. To investigate whether the KL-VS polymorphism could be associated with healthy aging and longevity in a Korean population, we analyzed genotype and allele frequencies of the KL-VS variant in a large Korean population sample. The KL-VS variant was not found in 874 Korean individuals. Thus, it is not possible to test its association to aging in the East Asian populations.

Klotho is a member of the glycosidase family 1 and a single-pass type-I transmembrane protein. It contains a signal sequence at the N-terminus and an extracellular domain, which is composed of two internal repeats, KL1 and KL2. These repeats exhibit 20-40% sequence homology to β-glycosidases (Kuro-o *et al.*, 1997). The protein translated from the Klotho gene exists in both secreted and membrane-bound forms (Matsumura *et al.*, 1998). The Klotho gene is expressed primarily in the prostate, placenta, and kidney (Shiraki-Iida *et al.*, 1998) and may play an important role in the regulation of calcium homeostasis (Nabeshima 2002), suppression of insulin and Wnt signaling (Liu *et al.*, 2007), amelioration of vascular endothelial dysfunction, increase of nitric oxide production, and reduction of elevated blood pressure (Saito *et al.*, 2000).

Klotho is an age-regulating protein. *KLOTHO*-deficient mice exhibit phenotypes resembling premature human aging (Kuro-o *et al.*, 1997). Deletion of the *KLOTHO* gene in mice leads to premature aging phenotypes including arteriosclerosis, osteopenia, and shortened life span. On the other hand, over-expression of this gene extends the lifespan of transgenic mice by 20-30% (Kurosu *et al.*, 2005).

The human *KLOTHO* gene is located on chromosome 13q12 and contains five exons. The expression "KL-VS polymorphism or variant" is used to describe a specific haplotype in a block of six SNPs, in perfect linkage disequilibrium. Of these six SNPs, rs9536314 (F352V) and rs9527025 (C370S) result in amino acid substitutions (Arking *et al.*, 2002). KL-VS refers to the V352 and S370 alleles of these SNPs and corresponds to a variant that shows reduced activity. The KL-VS polymorphism may alter the level of secreted Klotho form and the catalytic activities of Klotho protein (Arking *et al.*, 2002; Dubal *et al.*, 2014). The KL-VS variant spans exon 2 and its flanking sequence, and is common in Caucasians (Low *et al.*, 2005; Freathy *et al.*, 2006; Riancho *et al.*, 2007; Novelli *et al.*, 2008; Tsezou *et al.*, 2008; Invidia *et al.*, 2010; Nzietchueng *et al.*, 2011; Tavakkoly-Bazzaz *et al.*, 2011). KL-VS can influence *KLOTHO* gene expression *in vitro,* and it has been inconsistently associated with human longevity in European and American populations (Majumdar *et al.,* 2010). An association between KL-VS polymorphism and human longevity in Asian populations is unexplored and unknown. Here, we analyzed the genomic DNA of 874 healthy Koreans to investigate the frequency of the KL-VS variant of the *KLOTHO* gene and its possible association with human longevity in Koreans.

Participants were 874 healthy Korean controls (418 males and 456 females) consisting of 101 individuals ≤ 40 years of age, 671 individuals 41-79 years of age, and 102 individuals ≥ 80 years of age. The subjects were recruited during routine checkups at the Chuncheon Sacred Heart Hospital. Informed consent was obtained from all individuals. The study was approved by the Ethical Committee of Chonbuk National University. Genomic DNA was extracted from 200 μL whole blood using QIAamp^®^ DNA blood Mini Kit (QIAGEN, Valencia, CA, USA). Polymerase chain reaction (PCR) was performed with sense primer (5'-AGGCTCATGCCAAAGTCTGG-3') and antisense primer (5'-GTTTCCATGATGAACTTTTTGAGG-3'). After purification by using QIAquick^®^ Gel Extraction Kit (QIAGEN), the PCR products were directly sequenced with a model 3730 capillary electrophoresis sequencer (Applied Biosystems, Foster City, CA, USA). Statistical analyses were carried out using Statistical Analysis Software (SAS) version 9.3 (SAS Institute, Cary, NC, USA). The genotypes and allele frequencies of the KL-VS polymorphism were compared using the chi-square or Fisher's exact test.

The KL-VS polymorphism was not found in the Korean population sample (Table 1). The genotype and allele frequencies of the KL-VS polymorphism in the Korean population were significantly different from those previously reported in European and American populations. The present data are similar to data from Iranians but significantly different from data from Indian subjects (Table 1).

**Table 1:** Genotype and allele frequencies of the Klotho KL-VS variant sequence in various populations.

Population	Age, yr	Total	Genotype, n (%)			P-value	Allele, %		P-value	Reference
			WT/WT	WT/ VS	VS/VS		WT	VS		
UK Caucasian	29-35	1619	1158 (71.5)	409 (25.3)	52 (3.2)	-	84.2	15.8	-	Freathy *et al.,* 2006
Bohemian Czech	New Born	390	307 (78.7)	73 (18.7)	10 (2.6)	0.016	88.1	11.9	0.006	Arking *et al.,* 2003
	Elderly ≥ 75	415	308 (74.2)	103 (24.8)	4 (0.1)	0.040	86.6	13.4	0.078	
Baltimore Caucasian	New Born	420	309 (73.6)	100 (23.8)	11 (2.6)	0.652	85.5	14.5	0.347	
	Elderly ≥ 65	723	530 (73.3)	185 (25.6)	8 (1.1)	0.012	86.1	13.9	0.088	
Baltimore African American	New Born	226	156 (69.0)	58 (25.7)	12 (5.3)	0.259	81.9	18.1	0.213	
	Elderly ≥ 65	242	169 (69.8)	68 (28.1)	5 (2.1)	0.439	83.9	16.1	0.878	
American	Mean 54.3	143	95 (66.4)	44 (30.8)	4 (2.8)	0.350	81.8	18.2	0.302	Low *et al.*, 2005
US Caucasian	<35	332	241 (72.6)	85 (25.6)	6 (1.8)	0.390	85.4	14.6	0.425	Novelli *et al.*, 2008
	93-105	708	517 (73.0)	170 (24.0)	21 (3.0)	0.757	85.0	15.0	0.451	
Spanish	18-86 (mean 48.0)	438	330 (75.3)	104 (23.8)	4 (0.9)	0.021	87.2	12.8	0.025	Riancho *et al.,* 2007
Greek	Men (mean 61.5), Women (mean 65.8)	383	309 (80.7)	71 (18.4)	3 (0.8)	<0.001	90.0	10.0	<0.001	Tsezou *et al.,* 2008
Italian	Men <66, Women <73	463	348 (75.2)	103 (22.2)	12 (2.6)	0.300	86.3	13.7	0.113	Invidia *et al.,* 2010
	Men 66-88, Women 73-91	300	203 (67.7)	94 (31.3)	3 (1.0)	0.140	83.3	16.7	0.613	
	Men >88, Women >91	326	236 (72.4)	80 (24.5)	10 (3.1)	0.950	84.7	15.3	0.746	
French	25-88 (mean 57.5)	629	436 (69.3)	172 (27.4)	21 (3.3)	0.579	83.0	17.0	0.340	Nzietchueng *et al.,* 2011
Iranian	Mean 55	53	53 (100)	0 (0)	0 (0)	<0.001	100	0	<0.001	Tavakkoly-Bazzaz *et al.,* 2011
Indian	≤ 40	375	270 (72.0)	99 (26.4)	6 (1.6)	0.237	85.2	14.8	0.479	Majumdar *et al.,* 2010
	>40	199	140 (70.4)	53 (26.6)	6 (3.0)	0.911	83.7	16.3	0.801	
Korean	≤ 40 (mean 27.0)	101	101 (100)	0 (0)	0 (0)	<0.001	100	0	<0.001	This study
	40-79 (mean 62.9)	671	671 (100)	0 (0)	0 (0)	<0.001	100	0	<0.001	
	≥ 80 (mean 85.8)	102	102 (100)	0 (0)	0 (0)	<0.001	100	0	<0.001	

The KL-VS polymorphism was absent in a large Korean population sample. The KL-VS polymorphism is present in Caucasians, Americans, and Indians, but apparently not in Iranian, Korean, and Japanese populations (Majumdar *et al.*, 2010). The differences in the distribution of genotype and allele frequencies of this polymorphism suggest the possibility that the evolutionary distances are closer between Europeans and Americans than between Europeans and East Asians. The results obtained from SNP markers in human populations showed that European populations were closer to the Amerian populations than East Asians (Shriver *et al.*, 2004; Fazeli and Vallian, 2012). In our previous studies, we reported that the genotype frequencies of polymorphisms of certain genes are remarkably different between Koreans and Europeans (Jeong *et al.*, 2011, 2014).

Various polymorphisms of the *KLOTHO* gene including the KL-VS polymorphism and G-395A, G1110C, C1818T, and C2298T SNPs have been reported (Kawano *et al.*, 2002). Genetic association studies of the *KLOTHO* gene have been reported in sickle cell anemia, coronary artery disease (CAD), ischemic stroke, type 2 diabetes, hypertension, and hemodialysis (Arking *et al.*, 2003; Friedman *et al.*, 2009; Wang *et al.*, 2010). Among these *KLOTHO* polymorphisms, genetic association studies have been carried out to demonstrate an association between functional polymorphism of *KLOTHO* KL-VS and human aging and cognition, because the functional polymorphism of *KLOTHO* KL-VS was associated with modulation of its activity and trafficking of the protein (Dubal *et al.*, 2014). Some studies reported a positive correlation with longevity, but other studies did not report such an association (Di Bona *et al.*, 2014).

In conclusion, the KL-VS polymorphism of the *KLOTHO* gene was not found in our large Korean population sample, and hence it does not appear to be an effector of aging and human longevity in Koreans.
